# Lateral Femoral Cutaneous Nerve Decompression Guided by Preoperative Ultrasound Mapping

**DOI:** 10.7759/cureus.3652

**Published:** 2018-11-28

**Authors:** Jason Ellis, Julia R Schneider, Michael Cloney, Christopher J Winfree

**Affiliations:** 1 Neurosurgery, Lenox Hill Hospital, Donald and Barbara Zucker School of Medicine, New York, USA; 2 Neurosurgery, Brain Tumor Center, Lenox Hill Hospital, Hofstra Northwell School of Medicine, New York, USA; 3 Neurosurgery, Northwestern University, Chicago, USA; 4 Neurosurgery, Columbia University, New York, USA

**Keywords:** anterior superior iliac spine, meralgia paresthetica, peripheral neuropathy, nerve, ultrasound

## Abstract

Injury to the lateral femoral cutaneous nerve (LFCN) from compression or entrapment may result in meralgia paresthetica, a painful mononeuropathy of the anterolateral thigh. Surgical decompression of the LFCN may provide relief when conservative management fails. However, the considerable anatomic variability of this nerve may complicate surgical localization and thus prolong operative time. Herein, we report the use of preoperative high-resolution ultrasonography to map the LFCN in a patient with bilateral meralgia paresthetica. This simple, noninvasive imaging technique was seen to be effective at providing precise localization of the entrapped and, in this case, bilateral anatomically variant nerves. Preoperative high-resolution ultrasound mapping of the LCFN can be used to facilitate precise operative localization in the treatment of bilateral meralgia paresthetica. This is especially useful in the setting of suspected unusual nerve anatomy.

## Introduction

The lateral femoral cutaneous nerve (LFCN) arises from the lumbosacral plexus, travels through the pelvis, and may pass above, through, or under the inguinal ligament on its course into the superficial thigh. In most cases, the LCFN crosses medial to the anterior superior iliac spine (ASIS) and then divides into the anterior and posterior branches that innervate the anterolateral aspect of the respective thigh [1].

Injury to the LFCN causes meralgia paresthetica (MP), a painful mononeuropathy of the anterolateral thigh with an incidence of 4.3 per 100,000 person-years [2]. Compression or entrapment of the LFCN as it passes deep to, or through, the inguinal ligament are common causes of MP. Bilateral MP due to compressive injury is a known complication of prone positioning that typically presents early postoperatively and remits in the majority of cases without surgical intervention after approximately six months [3]. Decompressive surgery can provide relief when the pain is prolonged in duration, intractable, and conservative measures fail. However, the considerable anatomic variability of the LFCN may complicate surgical decompression, leading to difficult or inaccurate localization [4]. This may result in prolonged surgical exploration and even inadequate decompression if the nerve is not accurately identified.

Herein, we present a case of bilateral MP for which preoperative ultrasound guidance provided an extremely accurate anatomic localization, thus significantly reducing the intraoperative exploration. To our knowledge, this is the first report demonstrating the utility of a preoperative high-resolution ultrasound to guide definitive operative localization of both LFCNs. We propose a treatment paradigm to include precise ultrasound mapping before the LFCN decompression to maximize surgical efficacy and treatment outcome.

## Case presentation

A 63-year old male presented with a history of lumbar laminectomy and fusion seven months prior to his initial presentation. He recalls that two days following his prone lumbar operation he began experiencing severe bilateral pain along the respective anterolateral thigh. Lumbar magnetic resonance imaging (MRI) ruled out spinal nerve root-related pathology as causative and a definitive diagnosis of bilateral MP, secondary to LFCN compression during the prone spinal surgery, was rendered. Conservative measures with oral analgesics were initially recommended given the typically self-limited course of this pathology. Despite an increasing regimen, including NSAIDs, narcotics, and anti-neuropathic pain medications, the pain persisted over the course of several months. Additionally, traditional anatomically-guided local anesthetic injections were attempted without improvement in his symptoms. Thus, the patient elected to proceed with operative decompression after eight months of failed conservative therapy and worsening quality of life.

**Figure 1:**
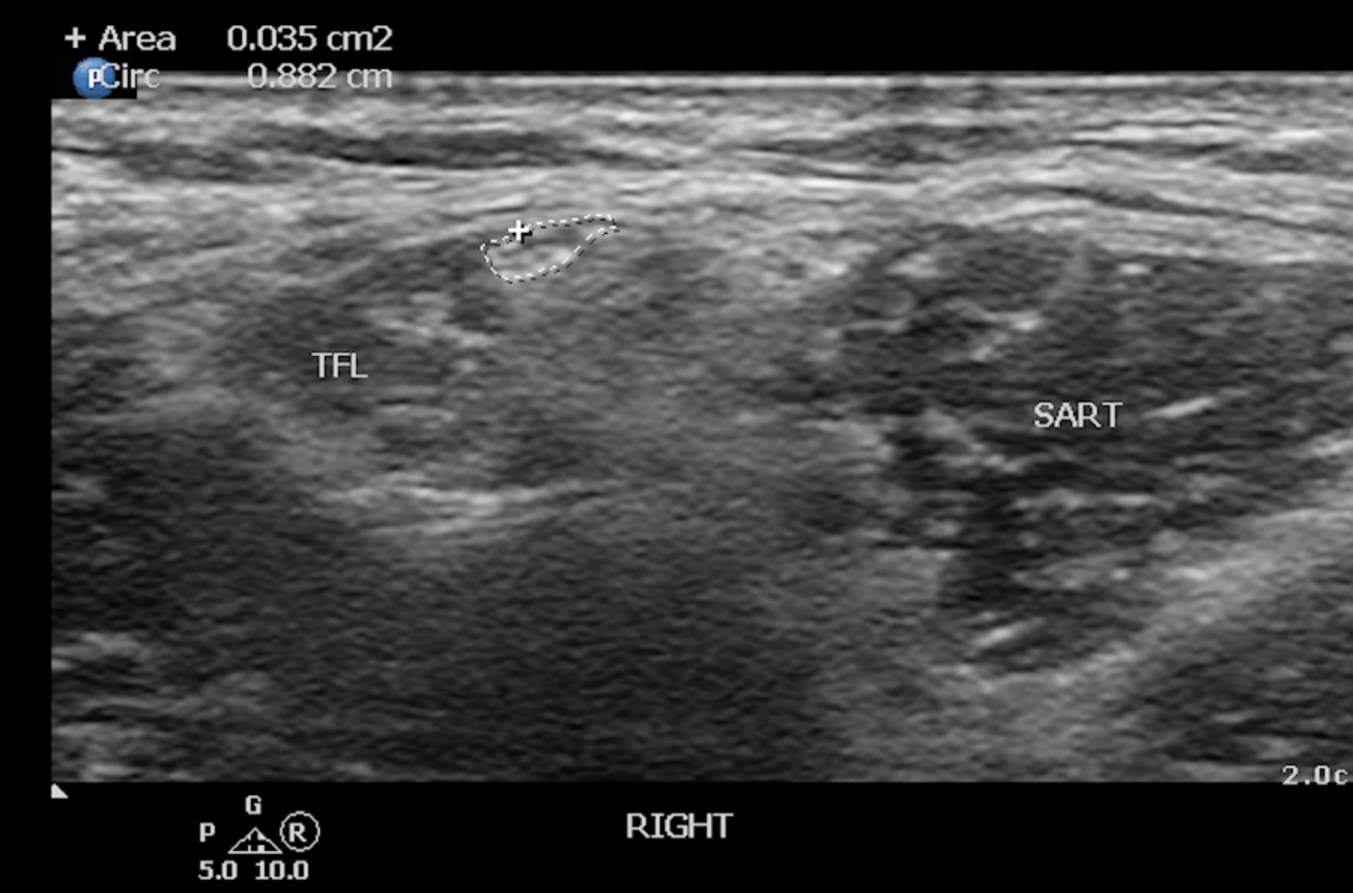
Ultrasound localization of the lateral femoral cutaneous nerve This ultrasound screen capture demonstrates the location of the right lateral femoral cutaneous nerve (circled) in the anterior thigh situated medial to the tensor fascia lata (TFL) and lateral to the sartorius (SART) muscle.

The history of failed local anesthesia to even temporarily alleviate symptoms suggested a possible non-classic nerve location and prompted preoperative ultrasound to outline the superficial course of the LFCN. The ultrasound technique utilized has been previously described in the setting of percutaneous injection guidance (Figure 1) [5].

**Figure 2:**
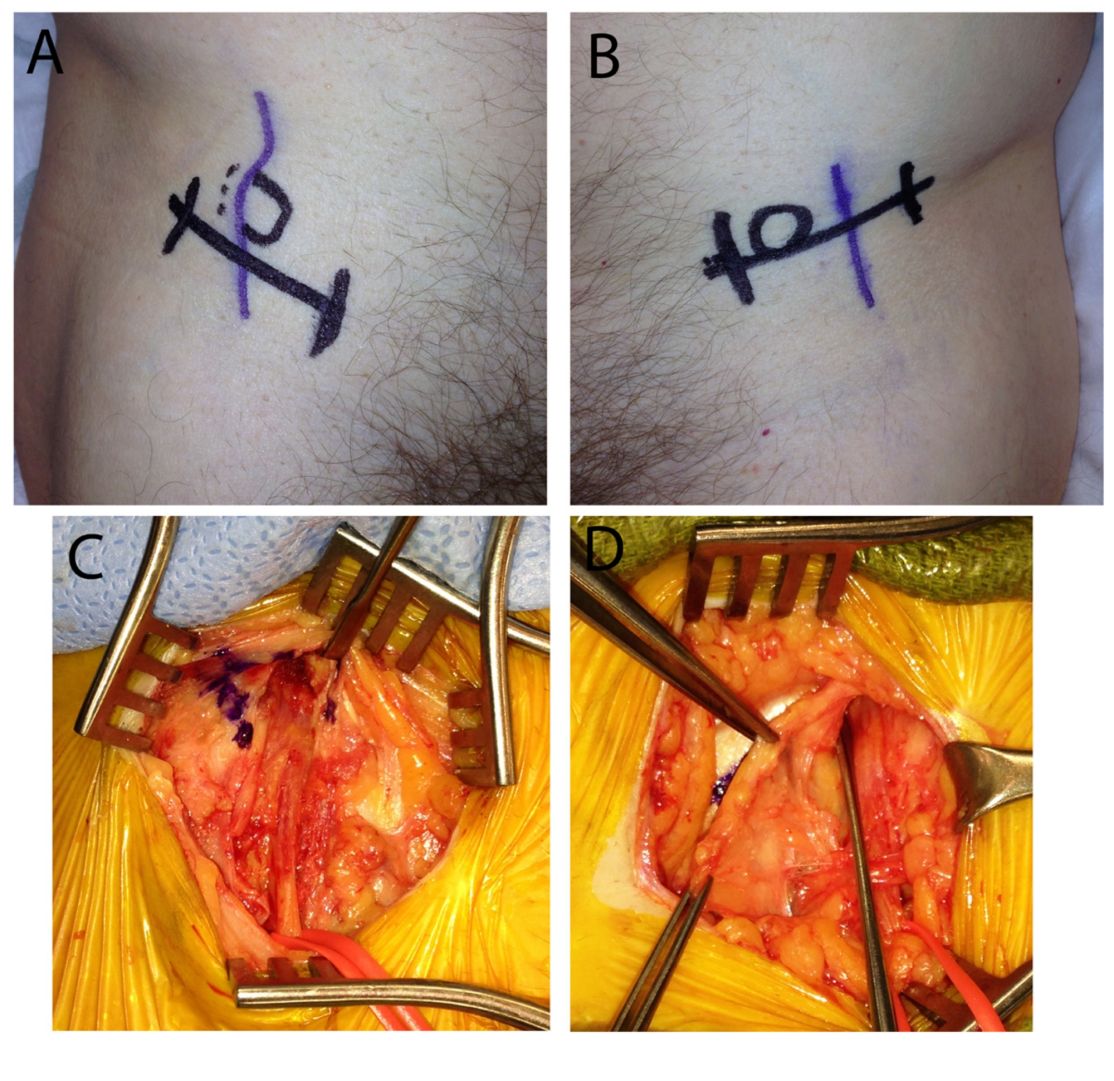
Variant course of the lateral femoral cutaneous nerves The course of the lateral femoral cutaneous nerve as it passes into the anterolateral thigh is demonstrated by the purple skin marking on both the right (A) and the left (B) legs. The intended incision is marked in black. It is notable that both lateral femoral cutaneous nerves pass in a non-classical location either at (A) or lateral (B) to the anterior superior iliac spine (circled). Intraoperative photographs demonstrate the close correlation between the surface markings and the LFCN after complete exposure on the right (C) and left sides (D). A probe is passed proximally along the nerve to confirm adequate decompression (D).

Skin markings in the inguinal region and upper thigh indicating the course of the LFCNs were made by the ultrasound technician prior to operative site preparation for surgery. Indeed, anatomically variant LFCNs were identified bilaterally on the preoperative ultrasound with neither nerve passing in a classic location medial to the anterior superior iliac spine (ASIS) (Figure 2). On the right, the ultrasound identified the LFCN passing directly over the ASIS, while on the left, the nerve was localized lateral to the ASIS. Oblique incisions were made centered over the marked LFCN locations identified by ultrasound. Both LFCN anatomical variants observed on preoperative ultrasound were confirmed after intraoperative dissection. Fascia overlying the nerves was opened to effect decompression. The fascial opening was confirmed adequate by ensuring the absence of entrapment along its course out of the pelvis into the thigh (Figure 2). The patient experienced immediate resolution of his preoperative pain and was discharged home on the same day without complication.

## Discussion

Ultrasonography has increasingly been used for peripheral nerve localization [5]. Ultrasound has proven particularly successful in the diagnosis of carpal tunnel syndrome for which it has a higher sensitivity (93% v. 67%) and equal specificity (86%) to MRI [6]. It has been suggested that ultrasound may be an alternative to electromyography (EMG) in some clinical settings [6-7]. Moreover, ultrasound has been demonstrated to be an effective means of attaining correct needle placement for a variety of blocks, including that for the LFCN [8-9]. Cadaveric studies comparing rates of successful LFCN localization using ultrasound guidance versus anatomic landmarks alone have demonstrated dramatically higher rates of successful needle-to-nerve contact when the ultrasound was utilized [10]. Although ultrasound has proven to be increasingly successful for noninvasive peripheral nerve visualization and for assessment of the fan type versus non-fan type of LCFN prior to total hip arthroplasty [11], to our knowledge, preoperative noninvasive ultrasound localization of the LFCN prior to decompressive surgery has not yet been described. Preoperative ultrasound-guided wire localization has been reported with preliminary feasibility; however, the approach is more invasive than using the high-resolution ultrasound alone, as the wire is placed around the nerve and the patient must be under anesthesia [12].

The LFCN demonstrates considerable anatomic variability, making ultrasound localization useful for cases such as ours. A cadaveric study of 200 patients revealed that 25.5% of patients have “abnormal” anatomy, and anatomic variants may be at higher risk for surgical injury [3, 13]. Most studies indicate that the LFCN passes medial to the ASIS at a mean distance of 1.4 - 2.9 cm [14-15]. It is notable that Hospodar et al. found no case in which the LFCN passed laterally to the ASIS in any of the 86 ilioinguinal dissections performed [16]. Variants, such as the one we report with the LFCN passing at or lateral to the ASIS, appear to be rare. In one report, this was suggested to occur in less than 3% of cases [15]. Awareness of such anatomical variations is critical to achieving successful decompression and neurolysis in such cases of MP [17-18]. Given the endless variations in the existence of the LFCN, preoperative ultrasound is deemed to be invaluable. 

Although MP is typically diagnosed clinically and remits spontaneously in the vast majority of cases [19], this etiologic variability makes imaging useful for cases where surgical intervention is being considered. Moreover, existing evidence suggests that ultrasound may be superior to MRI or EMG for diagnosis of certain peripheral nerve pathologies [20]. Although the clinical history, in this case, was classic for bilateral compression injury during prone position surgery, poor response to local anesthetic suggested the possibility of anatomically variant LFCNs. Indeed, preoperative ultrasound localization of the LFCNs allowed for precise, early localization of the rare anatomic variants reported.

## Conclusions

Preoperative high-resolution ultrasound can be a useful guide to the location of the lateral femoral cutaneous nerve when surgical intervention is being considered to treat meralgia paresthetica. Ultrasound may be even more useful in cases, such as ours, where a non-classic LFCN anatomic location is suspected based on a poor response to anatomically-guided local anesthetic delivery.
